# Evaluating the usefulness of breast strain elastography for intraductal lesions

**DOI:** 10.1007/s10396-020-01070-2

**Published:** 2021-01-03

**Authors:** Yumi Kokubu, Keiko Yamada, Masahiko Tanabe, Ayumi Izumori, Chieko Kato, Rie Horii, Shinji Ohno, Kiyoshi Matsueda

**Affiliations:** 1 Department of Ultrasound, The Cancer Institute Hospital of Japanese Foundation for Cancer Research, 3-8-31 Ariake, Koto-ku, Tokyo, 135-8550 Japan; 2 Department of Diagnostic Imaging, The Cancer Institute Hospital of Japanese Foundation for Cancer Research, Tokyo, Japan; 3 Department of Breast and Endocrine Surgery, The University of Tokyo Hospital, Tokyo, Japan; 4 Department of Breast Surgery, Takamatsu Heiwa Hospital, Takamatsu, Japan; 5 Department of Pathology, Saitama Cancer Center, Saitama, Japan; 6 Department of Pathology, The Cancer Institute of Japanese Foundation for Cancer Research, Tokyo, Japan; 7 Breast Oncology Center, The Cancer Institute Hospital of Japanese Foundation for Cancer Research, Tokyo, Japan

**Keywords:** Breast ultrasound, Strain elastography, Elasticity index (E-index), Elasticity ratio (E-ratio), Intraductal lesion

## Abstract

**Purpose:**

Strain elastography for imaging lesion stiffness is being used as a diagnostic aid in the malignant/benign discrimination of breast diseases. While acquiring elastography in addition to B-mode images has been reported to help avoid performing unnecessary biopsies, intraductal lesions are difficult to discriminate whether they are malignant or benign using elastography. An objective evaluation of strain in lesions was performed in this study by measuring the elasticity index (E-index) and elasticity ratio (E-ratio) of lesions as semi-quantitative numerical indicators of the color distribution of strain. We examined whether ductal carcinoma in situ (DCIS) and intraductal papilloma could be distinguished using these semi-quantitative numerical indicators.

**Methods:**

In this study, 170 ultrasonographically detected mass lesions in 162 cases (106 malignant lesions and 64 benign lesions)—in which tissue biopsy by core needle biopsy and vacuum-assisted biopsy, or surgically performed histopathological diagnosis, was performed—were selected as subjects from among 1978 consecutive cases (from January 2014 to December 2016) in which strain elastography images were acquired, in addition to standard B-mode breast ultrasonography, by measuring the E-index and E-ratio.

**Results:**

The cut-off values for E-index and E-ratio in the malignant/benign discrimination of breast lesions were determined to be optimal values at 3.5 and 4.2, respectively, based on receiver operating characteristic (ROC) curve analysis. E-index sensitivity, specificity, accuracy, and AUC value (area under the curve) were 85%, 86%, 85%, and 0.860, respectively, while those for E-ratio were 78%, 74%, 74%, and 0.780, respectively. E-index yielded superior results in all aspects of sensitivity, specificity, accuracy, and AUC values, compared to those of E-ratio. The mean E-index values for malignant tumors and benign tumors were 4.46 and 2.63, respectively, indicating a significant difference (*P* < 0.001). E-index values of 24 DCIS lesions and 25 intraductal papillomas were 3.88 and 3.35, respectively, which showed a considerably close value, while the false-negative rate for DCIS was 29.2%, and the false-positive rate for intraductal papilloma was as high as 32.0%.

**Conclusion:**

E-index in strain elastography yielded better results than E-ratio in the malignant/benign discrimination of breast diseases. On the other hand, E-index has a high false-negative rate and false-positive rate for intraductal lesions, a factor which should be taken into account when making ultrasound diagnoses.

## Introduction

Various types of elastography are currently installed in ultrasonic equipment, including strain elastography for applying manual compression vibrations or shear wave elastography for supplying vibration energy by radiation pressure irradiation [1]. Elastography is noninvasive and can be easily performed, with the usefulness for malignant/benign discrimination in regions other than the breast having been previously reported [2–4].

Malignant masses in the breast tend to be harder than the surrounding normal tissue. It has been reported that elastography, which makes use of the characteristics thereof and visualizes differences in strains, can add useful information to malignant/benign discrimination using it in addition to B-mode images, thus making it possible to avoid unnecessary biopsies [5–10].

The most common method for evaluating elastography in breast lesions is the five-point scoring system (Tsukuba score) [11]. Numerous studies have been conducted on the malignant/benign discrimination of breast lesions, using this score classification or the score distribution by histological type, and their usefulness has been reported [6, 12–15]. However, most previous studies do not include a sufficient number of intraductal lesions such as DCIS and intraductal papilloma. Itoh et al. and Yi et al. have reported that intraductal lesions are prone to false negatives or false positives according to the five-point scoring system [5, 11, 12]. On the other hand, actual elastography often reveals a complex color distribution, with a substantial number of cases that are difficult to score. For example, there are cases in which an intermediate pattern of score classification is indicated or there are cases that do not fit into the five patterns. Because scoring is subjectively determined by color distribution, there are some cases that are not suitable for performing a quantitative analysis.

E-index is a numerical index of the color distribution of strain in the region of interest (ROI) on elastography. On the other hand, E-ratio is the ratio to the E-index, respectively, measured from the lesion and surrounding subcutaneous fat tissue. The usefulness of E-index and E-ratio to distinguish malignant lesions from benign breast lesions has been reported [16, 17]. In this study, we measured the E-index and E-ratio of ultrasonographically detected mass lesions and reviewed their usefulness for malignant/benign discrimination by obtaining the cut-off values of these indices. Furthermore, we examined whether there is a numerical difference between E-index and E-ratio values of DCIS and intraductal papilloma.

## Patients and methods

### Patients

Elastography images were obtained for 1978 cases, using LOGIQ E9 equipment (GE Healthcare, Milwaukee, WI, USA), among 43,062 cases that underwent breast ultrasonography between January 2014 and December 2016. A histopathological diagnosis was made based on a core needle biopsy (CNB) and vacuum-assisted biopsy (VAB) on 216 lesions (208 cases) with mass images. Lesions that indicated a mass were chosen as the subjects for this study.

One hundred seventy lesions (162 cases) were selected as subjects, excluding those in which a tissue biopsy was performed prior to the examination (*n* = 17), those with recurrent lesions following surgery (*n* = 13), those difficult to discriminate (*n* = 11), and those who underwent preoperative chemotherapy (*n* = 5). This study excluded non-tumor lesions and those showing ductal abnormalities, a low echo in the breast, architectural distortion, and multiple microcysts [18].

The breakdown of the 170 lesions was 106 malignant lesions (76 invasive ductal carcinomas, 24 DCIS) and 64 benign lesions. The details of histological subtypes are shown in Table 1.

**Table 1:** Histological diagnosis

Pathologic diagnosis	Number of lesions	%
Malignant lesion (*n* = 106)		
Invasive ductal carcinoma	76	44.7
Ductal carcinoma in situ	24	14.1
Mucinous carcinoma	2	1.2
Invasive lobular carcinoma	2	1.2
Invasive micropapillary carcinoma	1	0.6
Secretory carcinoma	1	0.6
Benign lesions (*n* = 64)		
Fibroadenoma	29	17.1
Intraductal papilloma	25	14.7
Benign phyllodes tumor	4	2.4
Radial sclerosing lesion, sclerosing adenosis	2	1.2
Mastopathy	1	0.6
Tubular adenoma	1	0.6
Granulation tissue	1	0.6
Hamartoma	1	0.6

### Equipment

The LOGIQ E9 (GE Healthcare, Milwaukee, WI, USA) with the ML6-15-D broad-spectrum (4–15 MHz) linear transducer was used.

### Imaging methods

Strain elastography images were obtained after obtaining standard B-mode images. The following points were taken into account when acquiring images. Although the whole ROI box for all strain elastography images was set to include tumors, subcutaneous fat tissue, and the pectoralis major muscle, we prevented entry of the ribs or lungs. Furthermore, the area occupied by the tumor in the whole ROI box was set to be a quarter or less. For stable images, continuous minimal vibrations for 3 s or more were applied with a probe before obtaining the images. Because ultrasound settings and parameters can affect the elastography, they were all kept constant and fixed [19]. Image acquisition and ROI measurements were performed by a radiologist with over 20 years of experience in breast ultrasound.

### Measurement of E-index and E-ratio

E-index was measured by setting a measurement ROI inside the lesion on the B-mode screen. E-index is an index that quantifies the color distribution of a strain in the ROI. Because the average strain distribution obtained from the image is set at 1.0, lesions with an E-index greater than 0 and less than 1.0 indicate that the strain is greater than average, while lesions with an E-index greater than 1.0 indicate that the strain is less than average. The maximum E-index value is set at 6.0. After measuring E-index by setting the measurement ROI in the lesion, E-index of the subcutaneous fat tissue is measured by setting the measurement ROI in the subcutaneous fat tissue and, at the same time, E-ratio, which is the ratio of E-index of the lesion to E-index of the subcutaneous fat tissue, is calculated and displayed on the screen.

Depending on the size of the target lesion and the positional relationship with the fat tissue, breast tissue, ribs, and so forth around the lesion, it may be difficult to generate uniform strain when applying minimal vibrations. Therefore, there is a report that gives the depth of the lesion as the factor that has the greatest effect on elastography [20]. The measurement ROI of subcutaneous fat tissue was set at the same depth as the lesion, avoiding being directly above the lesion. The size of the measurement ROI for subcutaneous fat tissue was set as large as possible.

We acquired mean E-index and mean E-ratio for each histological type. We compared these mean values for invasive ductal carcinoma, DCIS, intraductal papilloma, and fibroadenoma using the *t *test. Mean E-index and E-ratio for malignant lesions and benign lesions were also compared by the *t* test, and the cut-off values for E-index and E-ratio were acquired using ROC curves.

### Statistical analyses

Statistical analyses were performed using the Microsoft Excel 2010 software program (Microsoft Corporation, Redmond, WA, USA) and JMP software program version 13.0 for Windows (SAS Institute Japan, Tokyo, Japan). Probability values (*P*) of each instance were calculated by *t* tests. *P* < 0.05 was taken to indicate statistical significance in all instances.

## Results

All cases were female and the average age was 52.4 years (22–86 years), with those having benign lesions averaging 47.6 years (22–86 years) and those having malignant lesions averaging 55.2 years (28–85 years), wherein a *t* test revealed that the age of those with malignant lesions tended to be significantly higher (*P* < 0.001). The average lesion size was 13.2 ± 8.47 mm, with malignant lesions averaging 13.3 ± 8.20 mm (4–50 mm) and benign lesions averaging 13.1 ± 8.96 mm (4–48 mm).

**Fig. 1:**
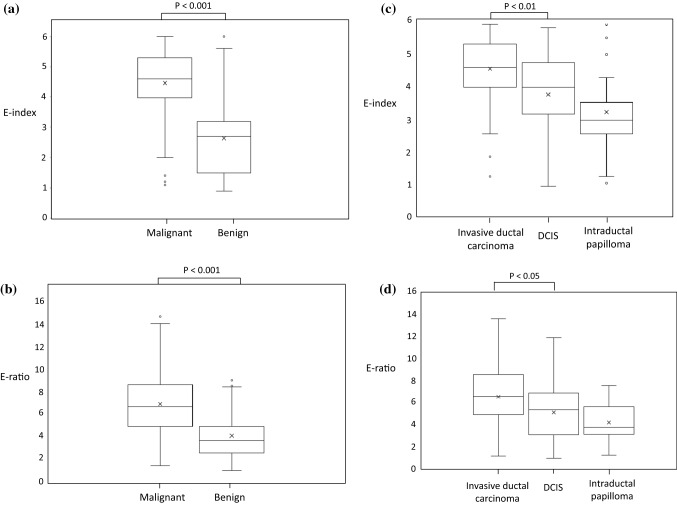
Mean E-index value. **a** The mean E-index values of 106 malignant (4.46 ± 1.08) and 64 benign (2.63 ± 1.22) lesions were significantly different (*P* < 0.001). **b** The mean E-ratio values of 103 malignant (6.23 ± 2.84) and 61 benign (3.49 ± 1.98) lesions were significantly different (*P* < 0.001). **c** The mean E-index values of 24 ductal carcinoma in situ (DCIS) (3.88 ± 1.20) and 76 invasive ductal carcinoma (4.66 ± 0.94) lesions were significantly different (*P *< 0.001). The mean E-index values of 24 DCIS and 25 intraductal papilloma (3.35 ± 1.26) lesions showed no significant difference (*P* = 0.14). **d** The mean E-ratio values of 24 DCIS (5.12 ± 2.52) and 73 invasive ductal carcinoma (6.46 ± 2.82) lesions were significantly different (*P* < 0.05). The mean E-ratio values of 24 DCIS and 25 intraductal papilloma (4.17 ± 1.55) lesions showed no significant difference (*P* = 0.14)

Mean E-index and E-ratio by histological type are shown in Table 2. Mean E-index values for malignant lesions (106 lesions) and benign lesions (64 lesions) were 4.46 (± 1.08) and 2.63 (± 1.22), respectively, and mean E-ratio values were 6.23 (± 2.84) and 3.49 (± 1.98), respectively, both indicating a statistically significant difference between malignant and benign lesions (*P* < 0.001; Fig. 1a, b). The mean E-index of 76 invasive ductal carcinoma lesions and 24 DCIS lesions was 4.66 (± 0.94) and 3.88 (± 1.20), respectively, indicating a significant difference (P < 0.01), and the mean E-ratio was 6.46 (± 2.82) and 5.12 (± 2.52), respectively, also indicating a significant difference (*P* < 0.05). Judging from the above, average E-index and E-ratio values for DCIS were significantly lower than those for invasive ductal carcinoma (Fig. 1c, d). The mean E-index and mean E-ratio for benign lesions were 2.13 (± 0.98) and 3.12 (± 2.13) for fibroadenoma (29 lesions), 3.35 (± 1.26) and 4.17 (± 1.55) for intraductal papilloma (25 lesions), and 2.98 (± 0.33) and 3.83 (± 1.12) for benign phyllodes tumor (4 lesions), respectively. E-index and E-ratio for intraductal papilloma showed the highest value among all benign lesions by histological type, and there was a significant difference in E-index and E-ratio between intraductal papilloma and all benign lesions excluding intraductal papilloma (*P* < 0.05). The mean E-index values for DCIS and intraductal papilloma, which are intraductal lesions, were very close, i.e., 3.88 and 3.35, respectively, with no significant difference between them (*P* = 0.14) (Fig. 1c). Similarly, the mean E-ratio values for DCIS and intraductal papilloma were very close, i.e., 5.12 and 4.17, respectively, with no significant difference between them (*P* = 0.14) (Fig. 1d).

**Table 2:** Mean E-index and E-ratio by histological type

Pathologic diagnosis	E-index	E-ratio
Malignant lesion (*n* = 106)	4.46	6.23
Invasive ductal carcinoma	4.66	6.46
Ductal carcinoma in situ	3.88	5.12
Mucinous carcinoma	5.10	9.35
Invasive lobular carcinoma	4.80	9.00
Invasive micropapillary carcinoma	5.10	9.40
Secretory carcinoma	1.20	1.70
Benign lesions (*n* = 64)	2.63	3.49
Fibroadenoma	2.13	3.12
Intraductal papilloma	3.35	4.17
Benign phyllodes tumor	2.98	3.83
Radial sclerosing lesion, sclerosing adenosis	2.90	4.10
Mastopathy	1.40	2.30
Tubular adenoma	1.50	1.10
Granulation tissue	1.10	1.30
Hamartoma	1.20	1.20

**Fig. 2:**
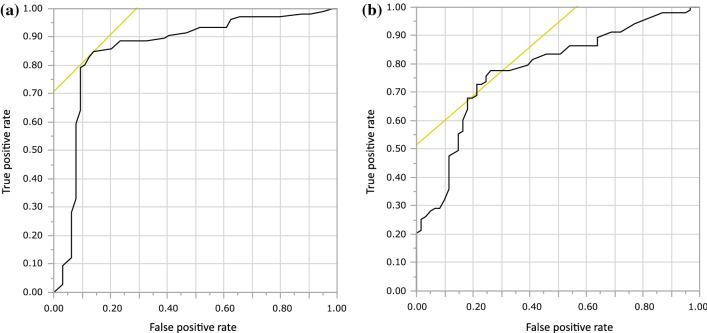
Receiver operating characteristic (ROC) curves for E-index and E-ratio. The sensitivity, specificity, and accuracy of E-index were 85%, 86%, and 85%, respectively, using a cut-off value of 3.5. The sensitivity, specificity, and accuracy of E-ratio were 78%, 74%, and 74%, respectively, using a cut-off value of 4.2. The areas under the curve for E-index and E-ratio were 0.86 and 0.78, respectively

ROC analysis was performed for the cut-off values for E-index and E-ratio (Fig. 2). E-index was analyzed based on 170 lesions and E-ratio based on 164 lesions. E-ratio could not be measured for six lesions (3.5% of the total). The reason for this was that there was almost no subcutaneous fat tissue on the same screen as the lesion. When the E-index cut-off value was set to 3.5, the sensitivity, specificity, and accuracy were 85%, 86%, and 85%, respectively. When the cut-off value for E-ratio was set to 4.2, the sensitivity, specificity, and accuracy were 78%, 74%, and 74%, respectively. The AUC of E-index and E-ratio was 0.86 and 0.78, respectively. Judging from the above, sensitivity, specificity, and accuracy were all superior for E-index as compared with E-ratio (Table 3).

**Table 3:** Sensitivity, specificity, and accuracy of E-index and E-ratio

	E-index	E-ratio
Cut-off point	3.5	4.2
Sensitivity (%)	85	78
Specificity (%)	86	74
Accuracy (%)	85	74
AUC	0.860	0.780

*AUC* area under the curve

**Fig. 3:**
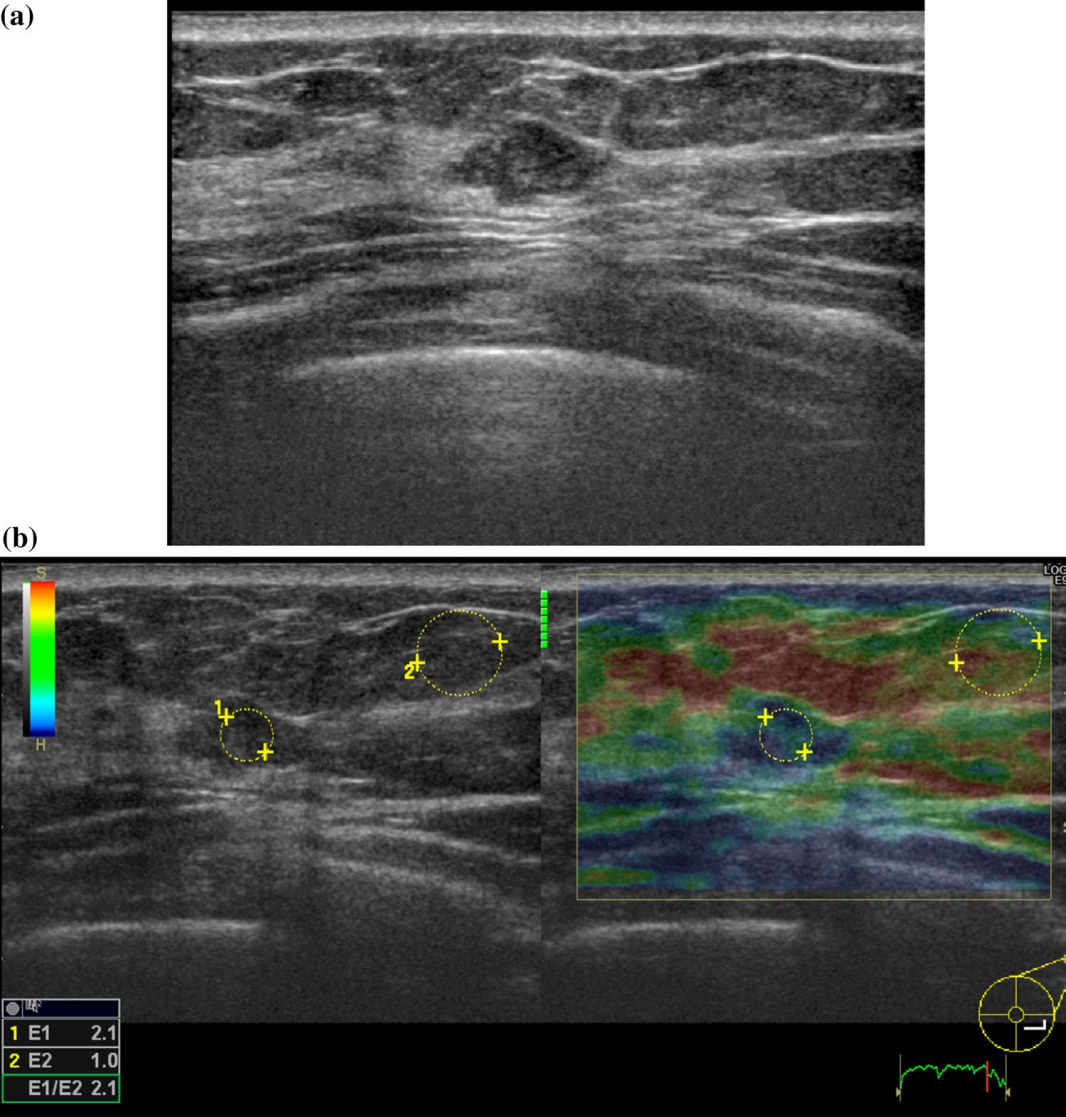
Ductal carcinoma in situ. **a** On B-mode ultrasound, there is a hypoechoic tumor that is relatively flat and slightly uneven inside. **b** The strain inside the tumor is uneven and has blue and green parts. E-index and E-ratio are both 2.1, which is much lower than the cut-off value

**Fig. 4:**
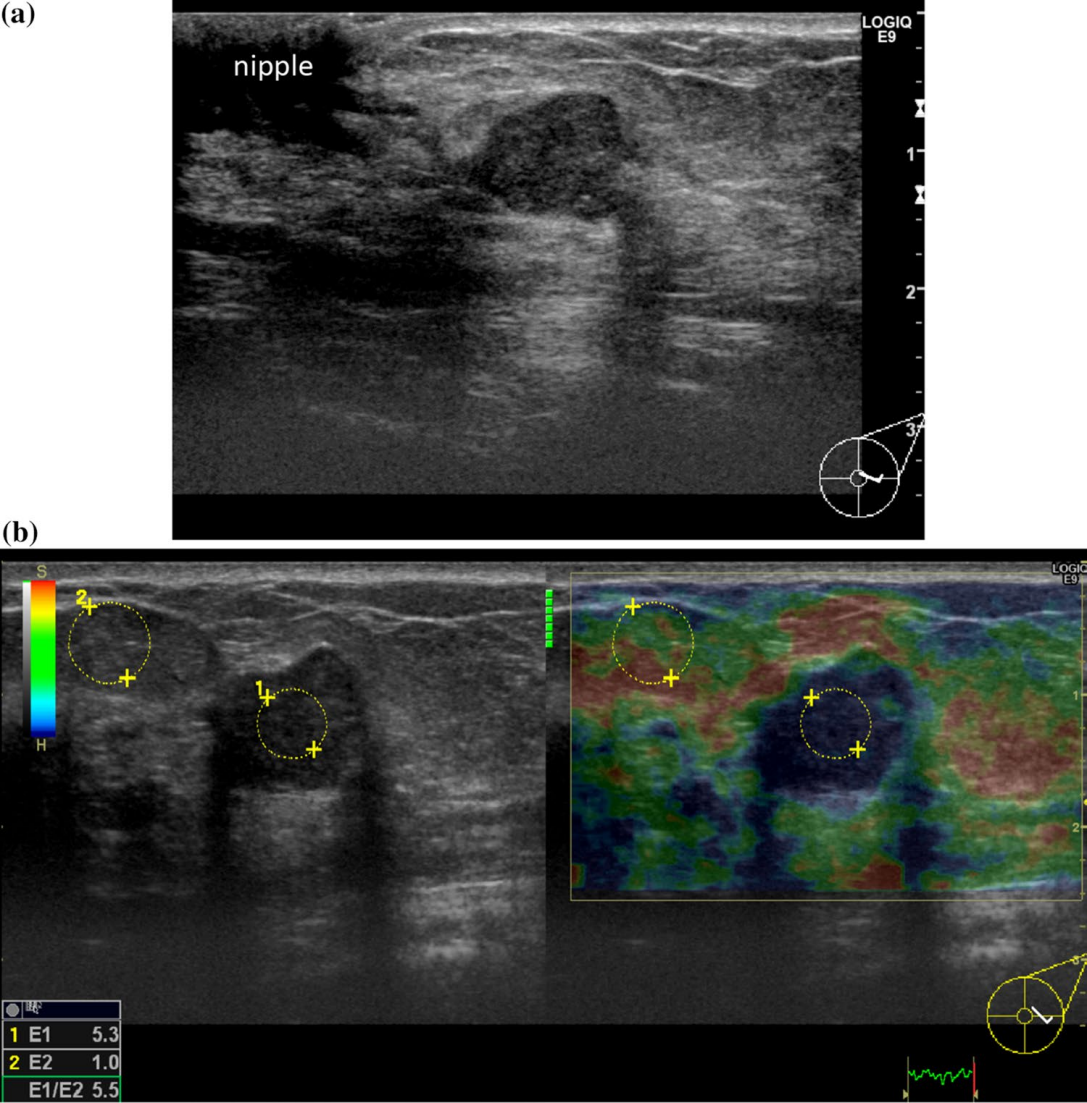
Intraductal papilloma. **a** On B-mode ultrasound, there is a polygonal hypoechoic tumor near the nipple and the back echo is intensified. **b** Strain is reduced throughout the tumor. E-index is 5.3 and E-ratio is 5.5, both of which are higher than the cut-off value

As for malignant/benign discrimination, with use of an E-index cut-off value of 3.5, 16 lesions (15.1%) were false negatives and nine lesions (14.1%) were false positives. The breakdown of false-negative lesions was eight invasive ductal carcinomas, seven DCIS, and one secretory carcinoma. False-negative lesions accounted for 10.5% of all invasive ductal carcinomas, and 29.2% of all DCIS, indicating they were very common with DCIS. Figure 3 is an example of a false-negative DCIS. The lesion was recognized as a flat hypoechoic mass on B-mode ultrasound, which is difficult to discriminate between malignant and benign. Elastography revealed a mixture of blue and green inside the lesion. Both E-index and E-ratio of the tumor were 2.1, which was much lower than the cut-off value and close to the mean value of benign lesions. On the other hand, the breakdown of the nine false-positive lesions (14.1%) was eight intraductal papillomas and one fibroadenoma. False-positive lesions accounted for 32.0% of all intraductal papillomas and 3.4% of all fibroadenomas, indicating that they were very common in intraductal papillomas. Figure 4 is an example of a false-positive intraductal papilloma. A low-echo mass that was difficult to discriminate between malignant and benign was found near the nipple on B-mode ultrasound. The whole tumor exhibited a blue color and was displayed as a tumor with reduced strain upon elastography. E-index was 5.3 and E-ratio was 5.5, both of which were significantly higher than the cut-off value and higher than the mean value of malignant lesions.

## Discussion

Elastography for evaluating the strain of a lesion is currently installed in most breast ultrasonography equipment. Elastography is broadly divided into strain elastography and shear wave elastography, with guidelines summarizing the features of each method having been prepared [1]. The five-point scoring system (Tsukuba score), in which the degree of strain is scored in five levels, is widely used in strain elastography. Furthermore, the usefulness of fat-to-lesion strain ratio (FLR), which quantifies the ratio of strain between the lesion and the surrounding fat tissue, has already been reported [15, 21, 22]. The FLR cut-off value of 4.3–5.0 is applied for malignant/benign discrimination in Hitachi equipment [18]. With the LOGIQ E9 equipment used in the present study, it was possible to quantify the color distribution of the strain in the ROI semi-quantitatively only by measuring E-index at the lesion. Based on the ultrasonographically detected mass lesions in this study in which the histopathologic diagnoses were made, the cut-off value of E-index for malignant/benign discrimination was 3.5. This figure is higher than the 3.15 reported by Matsuoka et al. [16]. The reason for this is considered to be due to the difference in target lesions. In the study by Matsuoka et al., the only malignant lesions targeted were invasive cancers, with DCIS not included. In our study, DCIS (24 lesions) accounted for 23% of all malignant lesions (106 lesions). Previous reports indicated that DCIS accounted for 6–28% of all malignant lesions [5, 6, 11–14], while DCIS accounted for a relatively high proportion in this study. Also, the ratio of malignant lesions (106 lesions) to all target lesions (170 lesions) was 62% in this study, which was higher than in previous reports (15–47%) [5, 6, 11–14].

We also examined E-ratio in this study, which is the ratio of E-index measured at the lesion to E-index measured based on the surrounding fat tissue. Comparing E-index and E-ratio, E-index was found to be superior to E-ratio in terms of sensitivity, specificity, and accuracy. Judging from this, it was considered sufficient to evaluate the strain of lesions by measuring E-index alone. Although FLR measurement is difficult in cases with extremely low subcutaneous fat tissue or cases with low subcutaneous fat tissue around the ROI, it is possible, even in such cases, to evaluate the strain with E-index of the lesion alone, which can be a simple and useful evaluation method.

In this study, we examined the E-index of histological types with many false negatives and false positives in elastography. The histological type with the most false negatives was DCIS (29.2%), while that with the most false positives was intraductal papilloma (32.0%). Although it has been reported in the past that DCIS is less likely to have a reduced strain compared to other malignant lesions, and many intraductal papillomas show reduced strain [12], there are few reports to date that have evaluated them numerically. The current study once again shows that DCIS and intraductal papillomas, which are intraductal lesions, make malignant/benign discrimination by elastography difficult.

Adding elastography to standard B-mode ultrasound examinations makes more accurate malignant/benign discrimination possible, allowing us to avoid unnecessary tissue biopsies. However, the false-negative rate is very high for DCIS, and the false-positive rate is high for intraductal papilloma. Based on the above findings, we believe that when planning for subsequent examinations, the possibility of DCIS even if there is no reduction in strain, and the possibility of benign lesions such as intraductal papillomas even if there is a reduction in strain, should be taken into consideration.

## Conclusion

E-index, which is a semi-quantitative index for breast ultrasound, was found to be useful for malignant/benign discrimination. The E-index cut-off value in breast tumors was 3.5, and the E-ratio cut-off value was 4.2. The sensitivity, specificity, and accuracy of E-index in malignant/benign discrimination of breast tumors were better than those for E-ratio. Mean E-index and E-ratio for DCIS and intraductal papilloma showed no significant difference. The histological type with the highest false-positive rate in malignant/benign discrimination using E-index was intraductal papilloma, while that with the highest false-negative rate was DCIS; therefore, close attention is warranted when performing strain elastography of the breast.
